# SBR-YOLO: context-position attention and adaptive feature fusion for student behavior recognition

**DOI:** 10.3389/fncom.2026.1804422

**Published:** 2026-03-18

**Authors:** Yunming Zhang

**Affiliations:** School of Marxism, Pingdingshan University, Pingdingshan, China

**Keywords:** attention mechanism, multi-scale feature fusion, smart classroom, student behavior recognition, YOLO

## Abstract

**Introduction:**

In classroom scenarios, student behaviors exhibit high intra-class variance and subtle inter-class differences, while complex backgrounds and severe occlusions pose significant challenges for accurate behavior recognition.

**Methods:**

SBR-YOLO is proposed as a student behavior detection framework for accurate and robust recognition in complex classroom environments. To address the challenges posed by visually similar behaviors and non-uniform spatial distributions of targets, a Behavior-aware Context-Position Attention module is designed, which leverages learnable positional encoding and inter-head interaction mechanisms to capture spatial dependencies among behavioral regions and enable discriminative feature learning. To handle substantial scale variations between front-row and back-row students, an Adaptive Spatial Feature Fusion mechanism is introduced at each output level of the neck, prior to the detection heads, which adaptively learns fusion weights for cross-scale feature integration. A Class-Aware Discriminative Loss function is further introduced to enhance fine-grained discrimination by enforcing intra-class compactness and inter-class separation constraints.

**Results:**

Experiments on SCB-Dataset3 demonstrate that SBR-YOLO achieves 74.2% mAP@50, representing a 6.4 percentage point improvement over the YOLOv8n baseline, with the parameter count increasing moderately from 3.0 M to 4.6 M.

**Discussion:**

Comprehensive ablation studies and comparative experiments with state-of-the-art methods confirm the effectiveness of SBR-YOLO for student behavior recognition in complex smart classroom environments.

## 1 Introduction

In recent years, the rapid advancement of artificial intelligence and computer vision technologies has driven the widespread deployment of intelligent monitoring systems in educational settings (Zhang et al., 2024; Badshah et al., 2023). Accurate understanding of student behavior in classroom environments supports objective assessment of student engagement, enables timely instructional adjustments, and facilitates the development of data-driven educational management systems (Dimitriadou and Lanitis, 2023; Liu et al., 2025). Compared with traditional manual observation methods, which are subjective, time-consuming, and difficult to scale across multiple classrooms simultaneously, automated behavior recognition systems provide a more consistent, efficient, and scalable solution for real-time classroom monitoring (Jia and He, 2024).

Object detection methods provide a well-established technical paradigm for behavior recognition tasks. Early approaches based on two-stage detectors, such as R-CNN (Girshick et al., 2014) and Faster R-CNN (Ren et al., 2015), achieved relatively high detection accuracy but suffered from high computational cost and slow inference speed, limiting their applicability in real-time scenarios. The introduction of the YOLO series (Hussain, 2024) and subsequent single-stage detection frameworks significantly improved detection speed while maintaining competitive accuracy, and these models have been widely validated across domains including industrial inspection, safety monitoring, and pedestrian detection (Yi et al., 2024; Markovic et al., 2024; Milanovic et al., 2024; Jovanovic et al., 2024; Zivkovic et al., 2025). Building on this foundation, single-stage detectors were further applied to classroom behavior recognition scenarios. Chen et al. (2023) integrated multi-head self-attention and enhanced feature extraction modules into a single-stage detector; although detection performance was improved, the attention module did not incorporate positional encoding, reducing the network’s ability to capture spatial dependencies among behavioral regions. Han et al. (2025) proposed WAD-YOLOv8 with deformable large-kernel attention and an improved neck structure; yet the neck still applied fixed weights during cross-scale feature fusion, which may limit detection performance when significant scale differences exist between front-row and back-row student targets. Cao et al. (2025) developed YOLO-AMM through joint optimization of attention, multi-scale fusion, and loss function design; even so, the loss function did not apply class-specific supervision for visually confusable category pairs, which may reduce classification accuracy for behaviors such as reading and writing. Sheng et al. (2025) proposed an improved single-stage detection model incorporating multi-scale large-kernel convolution and progressive feature optimization; despite improved feature extraction capability, the classification loss optimized only the predicted probability distribution without imposing intra-class compactness or inter-class separability constraints in the feature embedding space, limiting fine-grained discrimination among visually similar behaviors.

Despite the progress outlined above, existing methods for classroom behavior recognition still face three technical limitations that have not been adequately resolved. First, student behaviors in real classroom images exhibit high visual similarity within the same category; for example, the upper-body postures corresponding to reading and writing are nearly indistinguishable, and standard convolutional feature representations lack sufficient discriminability for fine-grained behavior recognition. Second, fixed-angle surveillance cameras in classrooms introduce significant scale variation between students in the front and back rows; existing feature pyramid networks aggregate multi-scale features using fixed weights that do not adapt to the spatial scale distribution of the input image, resulting in degraded recall for small and far-field targets. Third, standard cross-entropy loss applies uniform optimization pressure across all category pairs without imposing explicit constraints to separate visually confusable behavior classes, limiting classification accuracy under class-imbalanced and visually confusable training conditions.

To address the above limitations, SBR-YOLO is proposed as a student behavior recognition network built upon YOLOv8 with three targeted improvements. A Behavior-aware Context-Position Attention (BCPA) module is designed to capture spatial dependencies among behavior-relevant regions through learnable positional encoding and inter-head feature interaction, enhancing fine-grained discriminability for visually similar behaviors. An Adaptive Spatial Feature Fusion (ASFF) mechanism is introduced at each output level of the neck, prior to the detection heads, to replace fixed-weight feature aggregation with spatially adaptive cross-scale integration, improving detection performance for small and far-field targets with significant scale variation. A Class-Aware Discriminative Loss (CADL) function is formulated to jointly enforce intra-class compactness and inter-class separation in the feature space, strengthening the classification of confusable behavior categories under class-imbalanced and visually confusable training conditions.

The main contributions of this paper are as follows:

(1) SBR-YOLO is proposed as a unified student classroom behavior recognition framework that integrates behavior-aware attention, adaptive multi-scale feature fusion, and discriminative loss supervision within a single detection architecture.
(2) The BCPA module is designed to model behavior-relevant spatial dependencies through the combination of learnable positional encoding and inter-head attention interaction, providing more effective spatial context modeling for fine-grained discrimination of visually similar classroom behaviors compared to conventional attention mechanisms.
(3) The ASFF mechanism adjusts cross-scale feature aggregation weights in a spatially adaptive manner, effectively addressing the scale variation problem introduced by fixed-angle classroom cameras.
(4) The CADL function enforces explicit intra-class compactness and inter-class margin constraints, improving classification robustness for confusable behavior categories under class-imbalanced and visually confusable training conditions.

The remainder of this paper is organized as follows. Section 2 reviews related work. Section 3 describes the proposed SBR-YOLO framework in detail. Section 4 presents the experimental results and ablation studies. Section 5 concludes the paper.

## 2 Related work

### 2.1 Classroom behavior detection methods

Classroom behavior detection presents distinct challenges compared to general object detection, including severe inter-student occlusion, high target density, and significant scale variation introduced by fixed-angle overhead cameras. These characteristics necessitate detection architectures capable of extracting discriminative features across a wide range of target scales under fixed-viewpoint surveillance conditions Zhao and Zhu (2023) proposed CBPH-Net, which designs an efficient feature extraction module in the backbone to capture richer channel information and integrates PANet with coordinate attention in the neck to combine semantic and positional information. Rectangular boxes are further replaced with elliptical boxes when computing similarity between predicted and ground-truth annotations to reduce background interference. Wang et al. (2024) developed SBD-Net, incorporating a Focal Modulation module for multi-level feature fusion, a Dyhead detection head with three-dimensional attention across channel, spatial, and scale dimensions to enhance behavioral representation, and an ESLoss function to address the imbalance in behavior sample distribution. Wang et al. (2025) proposed SLBDetection-Net, designing a Learning Behavior-aware Attention mechanism to extract key features across multiple scales and constructing an LBA-Swin Transformer Block as the backbone encoder, enabling effective detection in both closed-set and open-set classroom scenarios. Nevertheless, these methods share a common limitation: the fixed-angle surveillance perspective inherent to classroom deployment introduces significant scale variation between front-row and back-row students, which existing feature extraction and fusion strategies fail to adequately address.

### 2.2 Attention mechanisms

Attention mechanisms improve object detection by enabling networks to assign adaptive importance weights to informative feature channels and spatial regions. Hu et al. (2018) introduced Squeeze-and-Excitation Networks (SENet), which recalibrate channel responses via global average pooling followed by a dimensionality-reduction bottleneck, modeling inter-channel dependencies with minimal parameter cost. Woo et al. (2018) proposed CBAM, which sequentially applies channel and spatial attention maps to intermediate feature representations, enabling the network to suppress irrelevant channels and spatial regions jointly. Wang et al. (2020) improved channel attention efficiency with ECA-Net, replacing the SENet reduction bottleneck with an adaptive-kernel one-dimensional convolution that captures local cross-channel interactions without information loss. Hou et al. (2021) proposed Coordinate Attention (CA), decomposing global pooling into horizontal and vertical 1D operations to embed precise directional position information into channel attention weights, improving localization of small and elongated targets. However, most existing mechanisms operate independently on either the channel or spatial dimension, limiting their capacity for joint cross-dimensional feature modeling. In multi-row classroom environments where behaviorally discriminative cues are spatially localized and frequently occluded, this limitation reduces the ability to discriminate visually similar postures across varying spatial locations.

### 2.3 Multi-scale feature fusion

Multi-scale feature fusion is essential for addressing the wide range of apparent target sizes in classroom surveillance images. Lin et al. (2017) proposed FPN, constructing a top-down pathway with lateral connections to propagate high-level semantic information to lower-resolution scales, substantially improving small-object detection. Tan et al. (2020) introduced BiFPN within the EfficientDet framework, assigning learnable weights to each feature fusion node and repeating the bidirectional pathway to enable more efficient cross-scale aggregation. Liu et al. (2019) proposed ASFF, a data-driven strategy that learns spatially varying scalar weights to filter conflicting information across pyramid levels before summation, suppressing scale inconsistency with negligible inference overhead. Although YOLOv8 adopts a PANet-based neck, it applies fixed-weight feature aggregation without content-aware weight assignment. In classroom scenarios where front-row and back-row students produce features with significant semantic and resolution gaps, static fusion strategies fail to selectively emphasize discriminative scale-specific information, resulting in degraded detection performance for small and far-field targets.

### 2.4 Loss functions for object detection

Accurate bounding box regression is critical in dense student detection scenes where targets are closely spaced and frequently occlude one another. Standard IoU loss provides no gradient when predicted and ground-truth boxes do not overlap, severely limiting convergence for non-overlapping predictions. Rezatofighi et al. (2019) proposed GIoU, incorporating the minimum enclosing box area as an additional penalty term to provide meaningful gradients in non-overlapping cases. Gevorgyan (2022) proposed SIoU, introducing an angular penalty term that considers the directional vector between predicted and ground-truth box centers, achieving faster convergence when predicted boxes deviate from the primary axes. Zhang et al. (2022) proposed EIoU, explicitly decomposing the shape penalty into independent width and height difference terms, enabling more precise geometric constraint than CIoU. Despite these advances, existing IoU-based loss functions focus exclusively on geometric localization and do not impose explicit feature-level constraints to separate visually confusable behavior categories such as reading and writing, leaving the joint challenge of fine-grained classification and accurate localization in crowded classroom scenes unresolved.

## 3 Methods

### 3.1 Overall architecture

**Figure 1:**
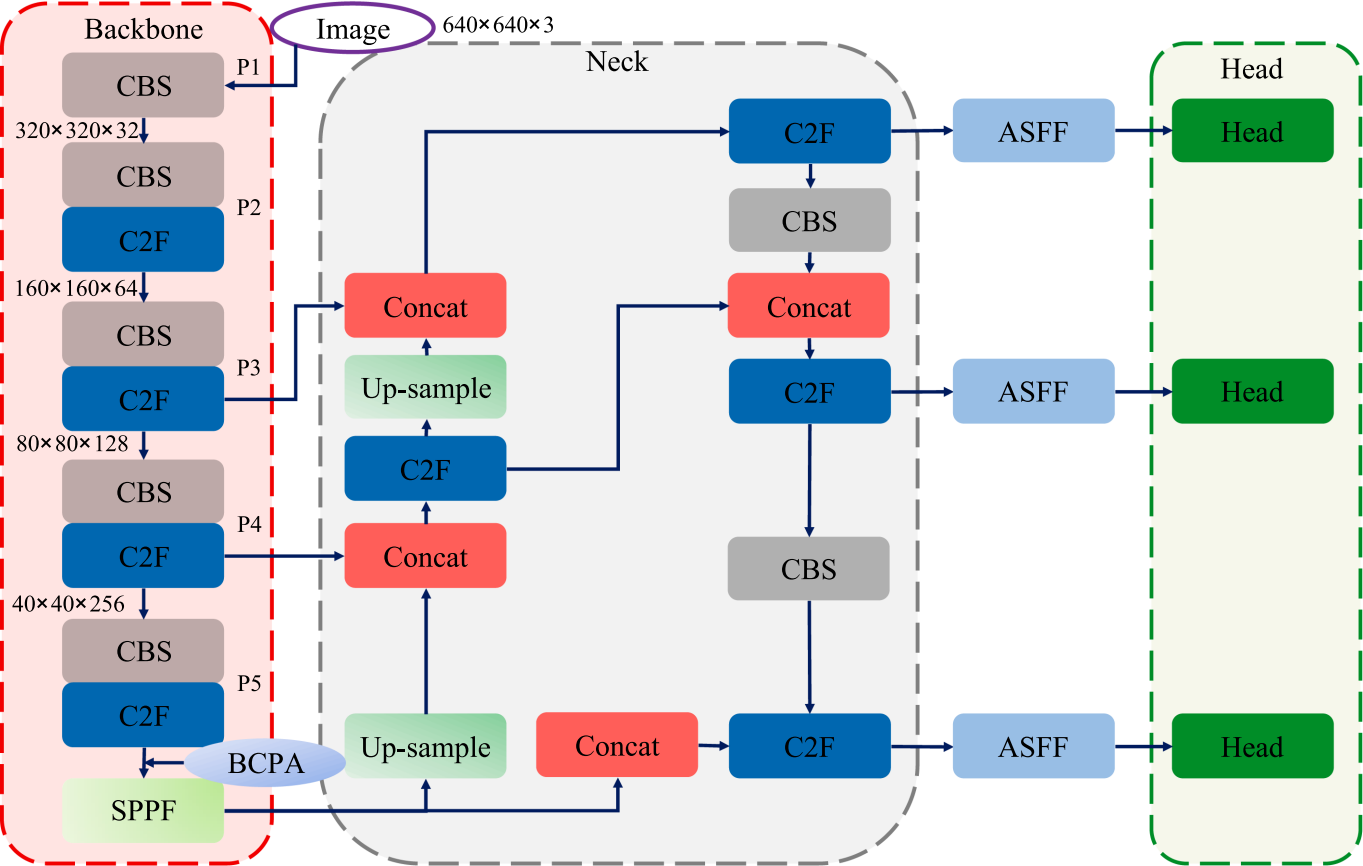
Overall architecture of the proposed SBR-YOLO framework.

**Figure 2:**
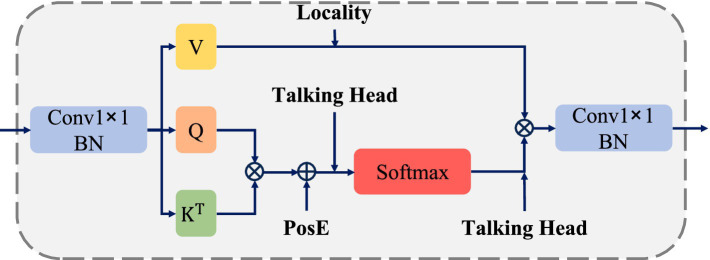
Structure of the behavior-aware context-position attention (BCPA) module.

As illustrated in Figure 1, the network architecture of SBR-YOLO is built upon the YOLOv8 framework, comprising three primary stages: a feature extraction backbone, a multi-scale feature aggregation neck, and task-specific detection heads. Three core components are integrated, namely BCPA, ASFF, and CADL. Specifically, the backbone adopts a CSPDarknet-based architecture, constructing a multi-scale feature pyramid through cascaded CBS and C2F modules, with the BCPA module embedded following the Spatial Pyramid Pooling layer to explicitly model spatial dependencies among behavioral regions via learnable positional encoding and inter-head interaction mechanisms. The neck utilizes an enhanced Path Aggregation Network structure to facilitate the bidirectional flow and fusion of deep semantic features and shallow spatial details; to handle significant target scale variations in classroom settings, ASFF modules are introduced at each output level of the neck, employing learnable spatially adaptive weights instead of fixed weights to achieve spatially adaptive integration of cross-scale features. The detection framework employs multi-scale parallel detection heads that predict bounding box coordinates, objectness scores, and class probabilities through decoupled convolution branches. During training, CADL replaces CIoU in the detection heads by jointly optimizing bounding box regression and feature-level discrimination through intra-class compactness and inter-class separation constraints, enhancing fine-grained discriminative capability for visually similar behaviors without incurring additional inference cost (Figure 2).

### 3.2 Behavior-aware context-position attention

Student behaviors in classroom environments exhibit non-uniform spatial distributions, with different behaviors occupying characteristic positions and regions within images. For instance, hand-raising typically occurs above the seating area with the arm extended upward, while reading and writing behaviors are both concentrated around desk regions with similar forward-leaning upper-body postures, making them particularly difficult to distinguish based on spatial location alone. Traditional attention mechanisms process spatial and channel information independently, struggling to model global contextual dependencies among behavioral regions. Furthermore, existing methods often neglect explicit positional encoding, despite the critical importance of spatial position information for accurate behavior recognition in classroom scenarios. To address these limitations, the Behavior-aware Context-Position Attention (BCPA) module is proposed, which achieves joint modeling of local details and global context through learnable positional encoding and inter-head interaction mechanisms, as illustrated in Figure 2.

The BCPA module adopts a Query-Key-Value (QKV) tri-branch structure as its foundational framework. Given an input feature map $F\in {\mathbb{R}}^{C\times H\times W}$ , the module first performs channel dimension adjustment through 1×1 convolution and batch normalization to obtain a refined feature representation $F_{r}\in {\mathbb{R}}^{C\times N}$ , where the spatial dimensions are flattened into sequence form with N=H×W denoting the total number of spatial positions. The channel dimension C is then uniformly partitioned into h=8 attention heads, each with per-head dimension d=C/h . The Query, Key, and Value branches are obtained via independent linear projections, as defined in Equation (1):


$$Q=W_{Q}F_{r},K=W_{K}F_{r},V=W_{V}F_{r}$$ (1)

where $W_{Q},W_{K},W_{V}\in {\mathbb{R}}^{C\times C}$ are learnable projection matrices, and $Q,K,V\in {\mathbb{R}}^{C\times N}$ . Each tensor is subsequently reshaped to h×d×N and then transposed to h×N×d for multi-head computation, such that $QK^{T}$ yields an attention score tensor of shape h×N×N .

Unlike conventional self-attention mechanisms, BCPA introduces learnable relative positional encoding $P\in {\mathbb{R}}^{N\times N}$ to explicitly model the spatial position information of behavioral targets. Each spatial position is identified by its row-column coordinate $p_{i}=(r_{i},c_{i})$ on the feature map. The coordinate difference $\Delta p=p_{i}-p_{j}\in {\mathbb{R}}^{2}$ between any two spatial positions $p_{i}$ and $p_{j}$ is fed into a compact MLP to produce the corresponding positional bias term $P_{\mathrm{ij}}$ , forming the complete positional encoding matrix P . The coordinate difference Δp is fixed for a given feature map resolution and is therefore consistent across input images of the same spatial dimensions. The contribution of the positional encoding lies in the trainable MLP, which learns position-behavior association patterns from these fixed coordinate differences during training, encoding a structural spatial prior of the classroom layout that is shared across all images.

Inspired by Talking-Head Attention, an inter-head interaction mechanism is introduced during attention weight computation to enhance information flow between different attention patterns. In traditional multi-head attention, each head computes independently without cross-head information exchange, limiting the representational capacity for complex behavioral patterns. BCPA addresses this by introducing learnable projection matrices $W_{\mathrm{pre}}$ and $W_{\text{post}}$ before and after Softmax normalization respectively, enabling information interaction and collaborative optimization among attention heads. Both $W_{\mathrm{pre}},W_{\text{post}}\in {\mathbb{R}}^{h\times h}$ operate across the head dimension of the attention score tensor of shape h×N×N , performing linear mixing among attention heads. The Pre-Softmax interaction operates on raw attention scores, adjusting the attention degree of different heads toward identical position pairs; the Post-Softmax interaction acts on normalized attention weights, further optimizing the final attention distribution. This dual inter-head interaction mechanism breaks the constraint of independent head computation, facilitating complementary fusion of multiple attention patterns.

Compared with standard multi-head self-attention, BCPA differs in two respects. First, the positional encoding is implemented as a learned MLP that takes the coordinate difference between two spatial positions as input, rather than using fixed sinusoidal encodings. This allows the module to learn position patterns specific to classroom images. Second, learnable projection matrices are applied before and after Softmax normalization to allow information exchange between attention heads, whereas standard multi-head attention computes each head independently.

Based on the above designs, the complete computational flow of the BCPA module is expressed in Equations (2) and (3):


$$A=\frac{W_{\mathrm{pre}}\cdot (QK^{T}+P)}{\sqrt{d}}$$ (2)

$$F_{\text{out}}={\text{Conv}}_{1\times 1}(V{\left(W_{\text{post}}\cdot \text{Softmax}(A)\right)}^{T})+F$$ (3)

where $QK^{T}$ computes the similarity matrix between queries and keys, P represents the learnable relative positional encoding, $\sqrt{d}$ serves as a scaling factor to stabilize gradients, $W_{\mathrm{pre}}$ and $W_{\text{post}}$ denote the projection matrices for Pre-Softmax and Post-Softmax inter-head interactions respectively, and the residual connection ensures stable gradient propagation.

The BCPA module offers several advantages. First, the learnable positional encoding enables the network to capture scene-specific position-behavior associations, such as recognizing that hand-raising behaviors are more likely to appear in the upper portion of images. Second, the QKV self-attention mechanism allows each spatial position to interact with all other positions, effectively modeling global contextual dependencies among behavioral regions, which is particularly important for distinguishing visually similar but semantically different behaviors. Third, the dual inter-head interaction mechanism enhances the expressive capacity of multi-head attention, enabling different attention heads to complementarily capture diverse behavioral patterns.

### 3.3 Adaptive spatial feature fusion

The YOLOv8 framework utilizes an enhanced PAN-FPN structure within its neck to facilitate hierarchical feature aggregation. However, direct fusion of multi-scale features may introduce semantic inconsistencies and representational conflicts across different pyramid levels. These challenges become more pronounced in student behavior recognition tasks due to cluttered classroom backgrounds and considerable scale variations among target instances. To address these limitations, an ASFF mechanism is introduced at each output level of the neck, prior to the detection heads, which adaptively learns fusion weights for cross-scale feature integration.

**Figure 3:**
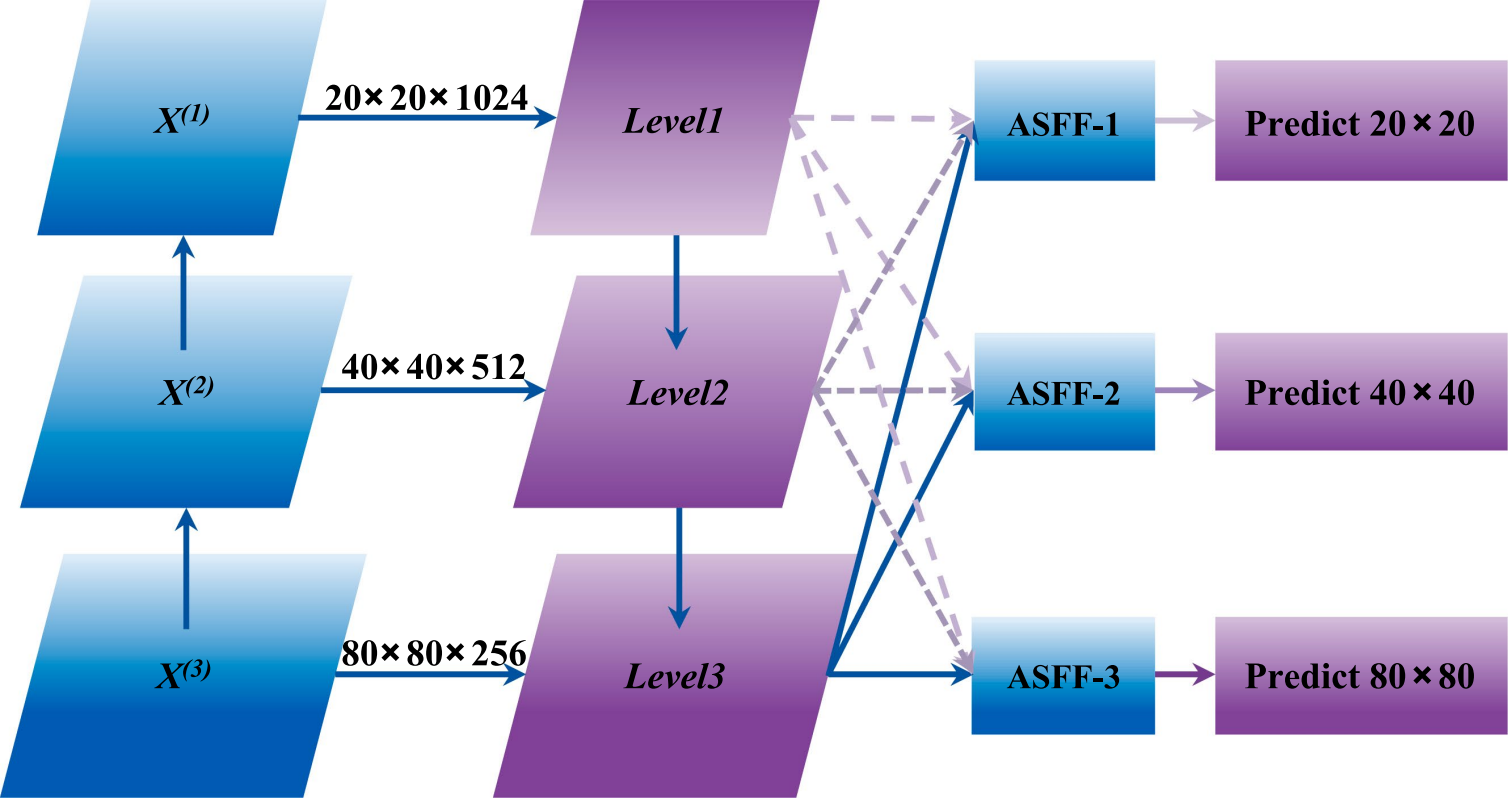
Architecture of the adaptive spatial feature fusion (ASFF) module.

As illustrated in Figure 3, the ASFF mechanism operates through two sequential stages: feature scale alignment and adaptive weighted aggregation. To achieve spatial and channel consistency across pyramid levels, scale-specific transformation operations are employed. For upsampling, 1 × 1 convolutions first reduce the channel dimension of the source feature map to match that of the target level, followed by bilinear interpolation with a scale factor of 2 to increase spatial resolution. For downsampling at a 1/2 ratio, 3 × 3 convolutions with stride 2 simultaneously reduce spatial dimensions and adjust channel counts. For 1/4 ratio downsampling, an additional max pooling layer with stride 2 is appended to the 1/2 downsampling pipeline. This parameterized transformation ensures that features from different scales are projected into a unified dimensional space.

The fused feature at each spatial location is computed through weighted summation of scale-aligned feature maps, as formulated in Equations (4)–(6):


$$y_{\mathrm{ij}}^{l}=\alpha_{\mathrm{ij}}^{l}\cdot x_{\mathrm{ij}}^{1\to l}+\beta_{\mathrm{ij}}^{l}\cdot x_{\mathrm{ij}}^{2\to l}+\gamma_{\mathrm{ij}}^{l}\cdot x_{\mathrm{ij}}^{3\to l}$$ (4)

where $x_{\mathrm{ij}}^{n\to l}$ denotes the feature vector transformed from level n to level l at spatial position (i,j) . The fusion weights $\alpha_{\mathrm{ij}}^{l},\beta_{\mathrm{ij}}^{l},\gamma_{\mathrm{ij}}^{l}\in [0,1]$ are spatially adaptive and learnable, determining the contribution of each scale-specific feature. These weights are normalized through softmax activation:


$$\alpha_{\mathrm{ij}}^{l}=\frac{e^{\lambda_{\alpha_{i,j}}^{l}}}{e^{\lambda_{\alpha_{i,j}}^{l}}+e^{\lambda_{\beta_{i,j}}^{l}}+e^{\lambda_{\gamma_{i,j}}^{l}}}$$ (5)

$$\alpha_{\mathrm{ij}}^{l}+\beta_{\mathrm{ij}}^{l}+\gamma_{\mathrm{ij}}^{l}=1$$ (6)

The weight parameters $\lambda_{\alpha_{i,j}}^{l},\lambda_{\beta_{i,j}}^{l},\lambda_{\gamma_{i,j}}^{l}$ are derived from the scale-aligned feature layers through 1×1 convolutions. Through this adaptive weighting scheme, ASFF strengthens the semantic representation of student behaviors across all feature pyramid levels, enabling effective cross-scale feature integration and enhancing discriminative capacity for behavior detection.

### 3.4 Class-aware discriminative loss

Student behaviors in classroom environments often exhibit high visual similarity, with representative examples such as reading and writing sharing nearly identical upper-body postures, posing significant challenges for accurate fine-grained classification. Standard detection losses address geometric regression and classification independently, without imposing explicit feature-level constraints to separate confusable behavior categories. The Class-Aware Discriminative Loss (CADL) is proposed to replace CIoU in the detection heads. CADL integrates bounding box regression with metric learning constraints, jointly optimizing geometric localization accuracy and feature-level discrimination among visually similar behavior categories. The complete training objective is formulated in Equation (7):


$${ℒ}_{\text{total}}={ℒ}_{\text{cadl}}+{ℒ}_{\mathrm{cls}}+{ℒ}_{\mathrm{dfl}}$$ (7)

where ${ℒ}_{\mathrm{cls}}$ denotes the binary cross-entropy classification loss and ${ℒ}_{\mathrm{dfl}}$ denotes the Distribution Focal Loss, both applied directly to the outputs of the detection heads following the default YOLOv8 training objective.

The intra-class compactness term in CADL follows the formulation of center loss. The difference is that CADL additionally includes a margin-based inter-class separation term, and both terms are applied to per-instance embeddings extracted from the detection head rather than to global image-level features as in the original center loss formulation.

This approach extends the center loss concept to the object detection domain with modifications tailored for multi-class behavior discrimination. Let $z_{i}\in {\mathbb{R}}^{D}$ denote the feature embedding for the i -th detection with ground truth class label $y_{i}$ , and let $\mu_{k}\in {\mathbb{R}}^{D}$ represent the running class centroid for class k computed across the training batch.

The intra-class compactness term encourages feature embeddings of the same class to cluster around their respective centroids, as expressed in Equation (8):


$$L_{\text{intra}}=\frac{1}{N}\sum_{i=1}^{N}\frac{∥z_{i}-\mu_{y_{i}}{∥}_{2}^{2}}{D}$$ (8)

where N denotes the number of samples and D represents the embedding dimension. This term penalizes dispersed class distributions, promoting compact cluster formation in the feature space.

The inter-class separation term promotes margin distance between different class centroids to enhance discriminability. Following contrastive learning principles, class centroids are constrained to maintain minimum separation, as defined in Equation (9):


$$L_{\text{inter}}=\frac{1}{K(K-1)}\sum_{j=1}^{K}\sum_{k\neq j}\max(0,m-∥\mu_{j}-\mu_{k}{∥}_{2})$$ (9)

where K denotes the number of classes and m is a margin hyperparameter empirically set to 2.0. This hinge-based formulation penalizes only centroid pairs within the margin threshold, avoiding excessive repulsion of already well-separated classes.

The complete CADL function combines both terms in Equation (10):


$${ℒ}_{\text{cadl}}={ℒ}_{\text{ciou}}+\lambda ({ℒ}_{\text{intra}}+\frac{{ℒ}_{\text{inter}}}{2})$$ (10)

The CIoU term ${ℒ}_{\text{ciou}}$ provides geometric regression supervision by jointly penalizing overlap, center distance, and aspect ratio deviation between predicted and ground-truth bounding boxes. The metric learning terms ${ℒ}_{\text{intra}}$ and ${ℒ}_{\text{inter}}$ operate on feature embeddings extracted from the penultimate layer of the detection head, enforcing intra-class compactness and inter-class separation to enhance discrimination among visually similar behaviors. The balance weight λ is empirically set to 0.5 to maintain comparable gradient magnitudes between the two components.

The class centroids $\mu_{k}$ are updated during training as exponential moving averages with momentum 0.9, as expressed in Equation (11):


$$\mu_{k}^{\left(t\right)}=\alpha \cdot \mu_{k}^{\left(t1\right)}+(1-\alpha )\cdot {\overset{ˉ}{z}}_{k}^{\left(t\right)}$$ (11)

where α=0.9 denotes the momentum coefficient and ${\overset{ˉ}{z}}_{k}^{\left(t\right)}$ represents the mean feature embedding of class k samples in the current batch.

The class centroids are initialized as zero vectors prior to training, i.e., $\mu_{k}^{\left(0\right)}=0\in {\mathbb{R}}^{D}$ . The per-batch class mean ${\overset{ˉ}{z}}_{k}^{\left(t\right)}$ is computed as the average feature embedding over all samples belonging to class k within the current training batch ${ℬ}_{k}^{\left(t\right)}$ as defined in Equation (12):


$${\overset{ˉ}{z}}_{k}^{\left(t\right)}=\frac{1}{|{ℬ}_{k}^{\left(t\right)}|}\sum_{i\in {ℬ}_{k}^{\left(t\right)}}z_{i}$$ (12)

where ${ℬ}_{k}^{\left(t\right)}$ denotes the set of samples whose ground-truth label is class k in the t -th training batch, and $|{ℬ}_{k}^{\left(t\right)}|$ denotes the cardinality of that set. When no sample of class k appears in a given batch, $\mu_{k}^{\left(t\right)}$ retains its value from the previous iteration.

## 4 Results and discussion

### 4.1 Datasets

SCB-Dataset3 (Yang and Wang, 2023) is adopted as the experimental benchmark, originating from video capture and frame-level annotation in real primary and secondary school teaching environments. SCB-Dataset3 encompasses 5,015 images with 25,810 annotated instances, covering three representative categories of student classroom behaviors: hand-raising, reading, and writing.

### 4.2 Evaluation metrics

The detection performance of the proposed algorithm is evaluated using seven metrics: Precision, Recall, mean Average Precision at IoU threshold 0.50 (mAP@50), mean Average Precision across IoU thresholds from 0.50 to 0.95 (mAP@50–95), F1 score, Parameters, and Floating-point Operations. The mathematical definitions of each metric are given in Equations (13)–(17) as follows:

Intersection over Union (IoU) quantifies the overlap between predicted bounding box $B_{p}$ and ground-truth bounding box $B_{\mathrm{gt}}$ , serving as the fundamental criterion for determining detection correctness:


$$\mathrm{IoU}=\frac{|B_{p}\cap B_{\mathrm{gt}}|}{|B_{p}\cup B_{\mathrm{gt}}|}$$ (13)

A prediction is considered correct when IoU exceeds the predefined threshold.

Precision reflects the proportion of correct detections among all predictions, while Recall characterizes the proportion of actual targets correctly detected:


$$P=\frac{N_{\mathrm{TP}}}{N_{\mathrm{TP}}+N_{\mathrm{FP}}}$$ (14)

$$R=\frac{N_{\mathrm{TP}}}{N_{\mathrm{TP}}+N_{\mathrm{FN}}}$$ (15)

where $N_{\mathrm{TP}}$ denotes the number of true positives, representing instances correctly identified as positive samples; $N_{\mathrm{FP}}$ denotes the number of false positives, representing negative samples incorrectly classified as positive; $N_{\mathrm{FN}}$ denotes the number of false negatives, representing positive samples that are missed.

Average Precision (AP) is computed as the area under the Precision-Recall curve, reflecting the detection performance for a single category:


$$\mathrm{AP}=\int_{0}^{1}P(r)\;\mathrm{dr}$$ (16)

Mean Average Precision averages the AP values across all categories, measuring the overall performance in multi-class detection tasks:


$$\mathrm{mAP}=\frac{1}{C}\sum_{i=1}^{C}{\mathrm{AP}}_{i}$$ (17)

where C represents the total number of detection categories.

Parameters denotes the total number of trainable parameters in the model, used to evaluate model complexity. Floating-point Operations (FLOPs) quantifies the computational complexity for a single forward pass, measured in giga-operations.

**Figure 4:**
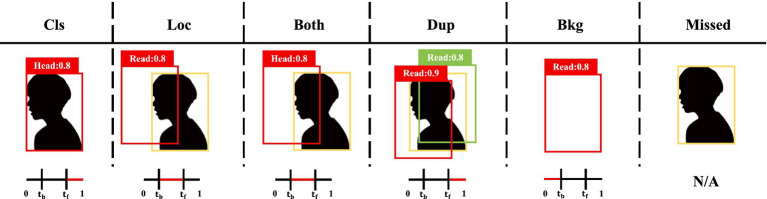
Visual representation of error categories within the TIDE evaluation protocol. Red boxes mark incorrect detections known as false positives, orange boxes signify ground-truth labels, and green boxes identify correct predictions referred to as true positives. The lower section illustrates the intersection over union alignment between predicted and ground-truth boxes, where the extent of overlap for each error type is highlighted in red.

The TIDE (Bolya et al., 2020) evaluation framework is adopted to analyze model errors across six categories: classification error (Ecls), localization error (Eloc), joint classification-localization error (Eboth), duplicate detection error (EDup), background error (Ebkgd), and missed detection error (Emiss). The metric IoUmax denotes the maximum IoU ratio between predicted bounding boxes and ground-truth annotations for the corresponding category. The foreground and background IoU thresholds, denoted as tf and tb, are set to 0.5 and 0.1, respectively. Detailed error type definitions are illustrated in Figure 4.

### 4.3 Experiment environment

All experiments were conducted on a unified software and hardware platform. The operating system was Ubuntu 22.04 LTS, with the programming environment based on Python 3.10.14. The deep learning framework was PyTorch 2.2.0, configured with CUDA 12.4 and cuDNN 9.1.0 to optimize GPU computational efficiency. The hardware configuration was as follows: the CPU was an Intel Core i9-14900K operating at 5.6 GHz with a 24-core 32-thread architecture; system memory was 64 GB DDR5 at 5600 MHz; the GPU was an NVIDIA GeForce RTX 4090D with 24 GB GDDR6X video memory.

The initial learning rate was set to 0.01 and adjusted dynamically via cosine annealing. The optimizer was Stochastic Gradient Descent with momentum 0.937 and weight decay 0.0005. The batch size was 16, training epochs were 200, and input images were resized to 640 × 640 pixels. Mosaic data augmentation was applied during the first 150 training epochs to enhance generalization. All models in the comparative experiments were trained from scratch without pretrained weights to ensure that performance differences were attributable solely to architectural design rather than initialization.

### 4.4 Performance comparison between SBR-YOLO and YOLOv8

The proposed model is evaluated on SCB-Dataset3, and the experimental results are compared against YOLOv8n as the baseline, as presented in Table 1. SBR-YOLO achieves consistent performance improvements over YOLOv8n across all evaluation metrics. The mAP@50 increases from 67.8 to 74.2%, a 6.4 percentage point improvement, with the parameter count increasing moderately from 3.0 M to 4.6 M. The TIDE error analysis provides detailed insights into the detection quality improvements across six error categories.

**Table 1:** Performance comparison between SBR-YOLO and YOLOv8n(%).

Method	Ecls	Eloc	Eboth	EDup	Ebkgd	Emiss	mAP@50	mAP@50–95	F1	Params (M)
SBR-YOLO	4.36	7.85	0.62	0.17	3.94	0.87	74.2	51.6	71.6	4.6
YOLOv8n	5.37	9.22	0.96	0.31	4.93	1.35	67.8	47.7	64.5	3.0

Specifically, the classification error (Ecls) decreases from 5.37 to 4.36%, suggesting that CADL enhances discriminative capability for visually similar behaviors through intra-class compactness and inter-class separation constraints. The localization error (Eloc) decreases from 9.22 to 7.85%, a 1.37 percentage point improvement, suggesting that the ASFF module addresses scale variations between front-row and back-row students through adaptive multi-scale feature fusion. The joint classification-localization error (Eboth) decreases from 0.96 to 0.62%, reflecting the synergistic effects of the proposed components. The duplicate detection error (EDup) decreases from 0.31 to 0.17%, suggesting improved localization precision that reduces redundant predictions for identical targets. The background error (Ebkgd) decreases from 4.93 to 3.94%, suggesting that the BCPA module suppresses background interference through position-aware attention mechanisms. The missed detection error (Emiss) decreases from 1.35 to 0.87%, a 0.48 percentage point improvement, confirming that SBR-YOLO exhibits enhanced detection capability for small-scale and occluded student targets.

**Figure 5:**
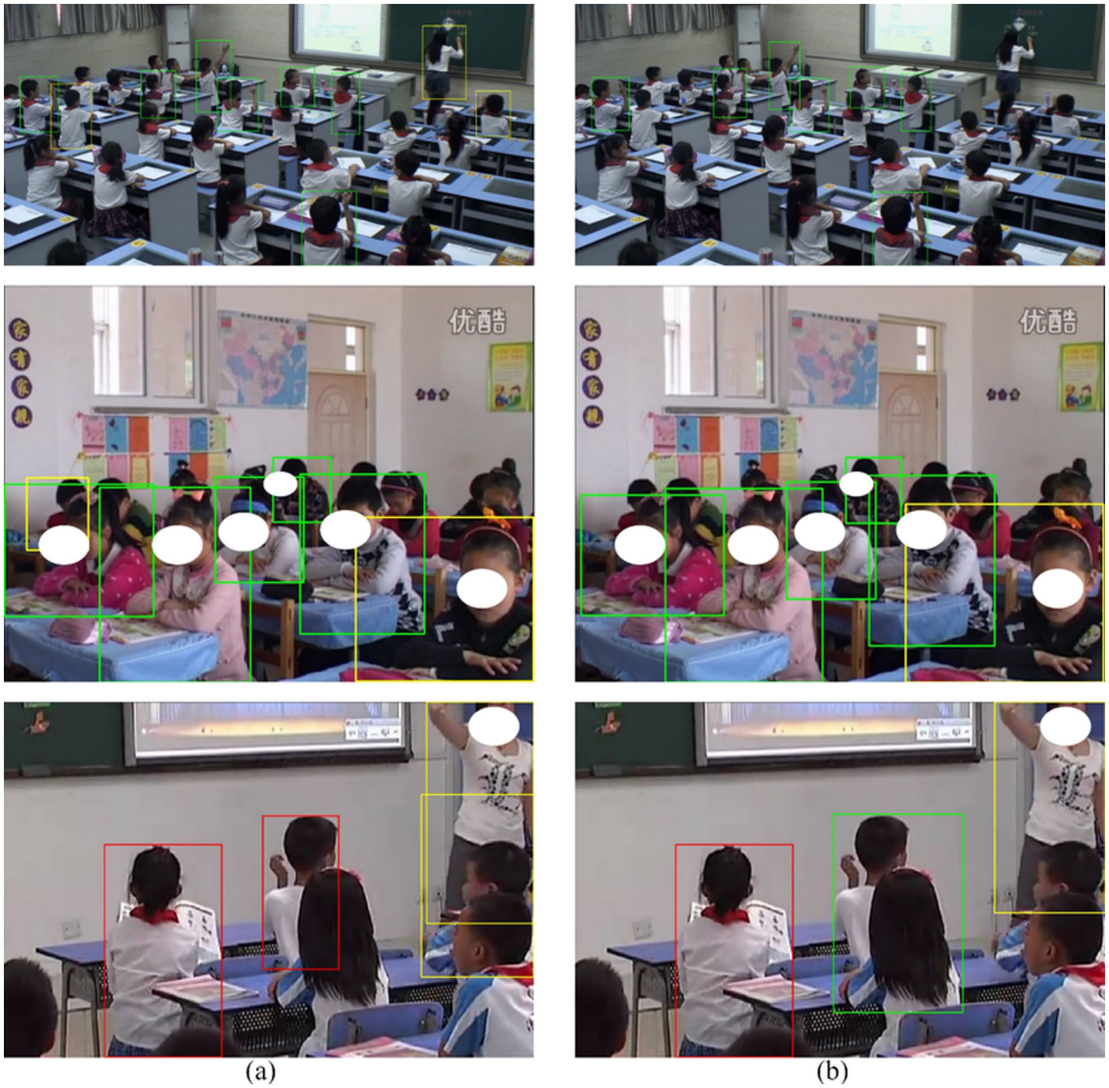
Visualization of detection results on the SCB-Dataset3: **(a)** YOLOv8n; **(b)** SBR-YOLO. Green, orange, and red bounding boxes represent true positives (TP), false positives (FP), and false negatives (FN), respectively.

The detection results of SBR-YOLO and YOLOv8n on SCB-Dataset3 are compared in Figure 5. The baseline model struggles to detect severely occluded and small-scale targets in back rows, frequently producing missed detections and false positives. In contrast, SBR-YOLO reduces the rates of missed detections and false positives, exhibiting enhanced robustness for occluded students and small-scale targets in distant seating areas. Occasional missed detections and false positives persist in certain extreme scenarios, primarily due to insufficient target resolution caused by long-distance imaging and severe occlusion. The quantitative error analysis and visualization results confirm that SBR-YOLO addresses detection challenges in complex classroom scenarios while maintaining computational efficiency.

### 4.5 Ablation study on module contributions

To validate the contribution of each proposed component, ablation experiments are conducted by progressively integrating BCPA, ASFF, and CADL into the YOLOv8n baseline. Table 2 presents results for all eight configurations ranging from the baseline to the full model.

**Table 2:** Ablation study results on SCB-dataset3 (%).

BCPA	ASFF	CADL	Precision	Recall	mAP@50	mAP@50–95	F1	Params (M)
			64.5	64.6	67.8	47.7	64.5	3.0
✓			68.7	67.9	70.6	49.5	68.3	3.5
	✓		66.9	66.8	69.4	48.9	66.8	4.3
		✓	67.2	66.3	69.1	48.3	66.7	3.0
✓	✓		70.5	69.6	72.3	50.8	70.0	4.6
✓		✓	70.1	69.2	71.9	50.3	69.6	3.5
	✓	✓	68.6	68.1	70.8	49.6	68.3	4.3
✓	✓	✓	72.4	70.8	74.2	51.6	71.6	4.6

The baseline YOLOv8n achieves 67.8% mAP@50 with 3.0 M parameters. When integrated independently, BCPA yields the largest single-component improvement of 2.8 percentage points, elevating mAP@50 to 70.6%. This confirms that learnable positional encoding and inter-head interaction mechanisms effectively model global contextual dependencies and enable discriminative feature extraction for behavior-relevant regions. ASFF contributes a 1.6 percentage point improvement by adaptively aggregating multi-scale features through learned spatial weights, addressing the scale variation between front-row and back-row targets. CADL achieves a 1.3 percentage point gain without introducing additional parameters, demonstrating that jointly optimizing geometric regression and feature-level discrimination enhances inter-class separability for visually similar behaviors.

The pairwise combinations reveal synergistic effects among components. BCPA+ASFF achieves 72.3% mAP@50, indicating that attention-refined features benefit from adaptive cross-scale aggregation. BCPA+CADL attains 71.9% mAP@50, suggesting improved feature discriminability under joint spatial-semantic supervision. ASFF+CADL reaches 70.8% mAP@50, demonstrating complementary effects between feature-level fusion and loss-level constraints.

### 4.6 Comparative analysis of attention mechanisms

To evaluate the proposed BCPA module, comparative experiments are conducted against representative attention mechanisms including SE, ECA, CA, and CBAM. All modules are integrated at identical positions within the YOLOv8n backbone to ensure a fair comparison. Table 3 presents the quantitative results.

**Table 3:** Comparison of attention mechanisms (%).

Attention type	Precision	Recall	mAP@50	mAP@50–95	F1
Baseline	64.5	64.6	67.8	47.7	64.5
SE	65.6	65.3	68.5	48.2	65.4
ECA	66.1	65.8	68.9	48.6	65.9
CA	67.4	66.5	69.7	49.4	66.9
CBAM	67.1	66.8	69.3	48.9	66.9
BCPA(Ours)	68.7	67.9	70.6	49.5	68.3

Channel-wise attention mechanisms demonstrate limited performance for behavior recognition. SE and ECA achieve marginal improvements of 0.7 and 1.1 percentage points respectively, as global channel recalibration fails to capture fine-grained spatial patterns essential for distinguishing visually similar postures. CA incorporates coordinate encoding into channel attention, yielding 69.7% mAP@50 through explicit spatial position modeling. CBAM integrates channel and spatial attention sequentially, achieving 69.3% mAP@50 via dual-dimension feature refinement.

BCPA outperforms all competing mechanisms with 70.6% mAP@50, surpassing CBAM by 1.3 percentage points and CA by 0.9 percentage points. The module leverages learnable relative positional encoding to capture spatial dependencies among behavioral regions, enabling position-behavior association learning; the inter-head interaction mechanism facilitates cross-head information exchange, enhancing the representational capacity for complex behavioral patterns. These results indicate that behavior-aware spatial modeling is more effective than generic attention mechanisms for student behavior recognition tasks.

### 4.7 Evaluation of loss function variants

To evaluate the proposed CADL, comparative experiments are conducted against commonly adopted loss functions for object detection (Zhang et al., 2023; Tong et al., 2023). All experiments use identical network architecture and training configurations to ensure a fair comparison. Table 4 presents the results.

**Table 4:** Comparison of loss functions (%).

Loss function	Precision	Recall	mAP@50	mAP@50–95	F1
CIOU	64.5	64.6	67.8	47.7	64.5
GIoU	64.1	64.3	67.2	47.1	64.2
EIoU	65.2	65.1	68.3	48.0	65.1
SIoU	65.8	65.6	68.6	48.4	65.7
Inner-IoU	65.4	65.2	68.1	47.8	65.3
WIoU	66.3	65.7	68.7	48.5	66.0
CADL	67.2	66.3	69.1	49.2	66.7

GIoU yields 67.2% mAP@50, a 0.6 percentage point degradation relative to CIoU, attributable to the minimum enclosing box penalty becoming less effective under dense target distributions. EIoU and SIoU achieve 68.3 and 68.6% mAP@50, respectively, by enhancing geometric constraints in bounding box regression. Inner-IoU achieves 68.1% mAP@50 by introducing an auxiliary bounding box to compute a scaled IoU, improving regression stability for densely distributed targets. WIoU attains 68.7% mAP@50 through a dynamic gradient weighting strategy. CADL achieves the highest mAP@50 of 69.1%, outperforming WIoU by 0.4 percentage points and CIoU by 1.3 percentage points. Unlike conventional IoU-based losses that focus solely on geometric localization, CADL jointly optimizes bounding box regression and feature-level discrimination through intra-class compactness and inter-class separation constraints, addressing both localization and fine-grained classification simultaneously. The intra-class compactness term penalizes the deviation of instance embeddings from their respective class centroids, promoting compact cluster formation within each behavior category, while the margin-based inter-class separation term enforces a minimum margin between class centroids in the embedding space, enhancing inter-class separability for confusable categories such as reading and writing.

### 4.8 Comparison with state-of-the-art methods

Comparative experiments are conducted against state-of-the-art detection methods spanning two-stage detectors, single-stage detectors, and recent YOLO variants to evaluate SBR-YOLO. Table 5 presents the quantitative results on SCB-Dataset3.

**Table 5:** Performance comparison with state-of-the-art methods on SCB-dataset3 (%).

Method	Precision	Recall	mAP@50	mAP@50–95	F1	Params(M)	FLOPs(G)
Faster R-CNN	58.3	61.2	62.7	43.6	59.7	41.8	134.5
SSD	54.6	56.8	57.3	39.2	55.7	26.3	62.7
RetinaNet	60.2	62.5	64.1	44.5	61.3	36.5	97.2
YOLOv3-tiny	62.7	57.9	61.4	42.3	60.2	12.1	19.0
YOLOv5n	57.6	64.8	64.2	44.7	61.0	2.5	7.2
YOLOv6n	61.0	65.1	66.9	46.8	63.0	4.2	11.9
YOLOv8n	64.5	64.6	67.8	47.7	64.5	3.0	8.0
YOLOv9c	69.0	67.5	70.4	50.1	68.2	25.5	103.7
YOLOv10n	63.9	61.3	65.4	45.3	62.6	2.7	8.4
YOLOv11n	61.7	63.6	65.6	45.6	62.6	2.5	6.3
YOLOv12n	59.2	63.2	63.7	43.9	61.1	2.5	6.5
SBR-YOLO (Ours)	72.4	70.8	74.2	51.6	71.6	4.6	11.9

Among conventional detectors, Faster R-CNN achieves 62.7% mAP@50, with 41.8 M parameters and 134.5G FLOPs, rendering it impractical for real-time deployment. SSD and RetinaNet yield 57.3 and 64.1% mAP@50 respectively, indicating that generic anchor-based architectures struggle with the high intra-class variance in student behaviors.

Within the YOLO family, YOLOv8n serves as a strong baseline with 67.8% mAP@50, 3.0 M parameters, and 8.0G FLOPs. Recent variants including YOLOv10n, YOLOv11n, and YOLOv12n underperform YOLOv8n, achieving 65.4, 65.6, and 63.7% mAP@50 respectively, suggesting that general architectural advances do not necessarily translate to improved performance on domain-specific recognition tasks. YOLOv9c achieves 70.4% mAP@50 but incurs higher computational costs, with 25.5 M parameters and 103.7G FLOPs.

SBR-YOLO achieves the highest mAP@50 of 74.2%, with 72.4% precision and 70.8% recall. Compared to YOLOv8n, SBR-YOLO improves mAP@50 by 6.4 percentage points with a moderate parameter increase from 3.0 M to 4.6 M. Compared to YOLOv9c, SBR-YOLO achieves 3.8 percentage points higher mAP@50 while requiring only 18.0% of the parameters and 11.5% of the FLOPs. These results indicate that task-specific architectural designs incorporating behavior-aware attention, adaptive multi-scale fusion, and discriminative metric learning achieve a favorable balance between accuracy and efficiency compared to general-purpose detection frameworks.

### 4.9 Visualization of detection results

**Figure 6:**
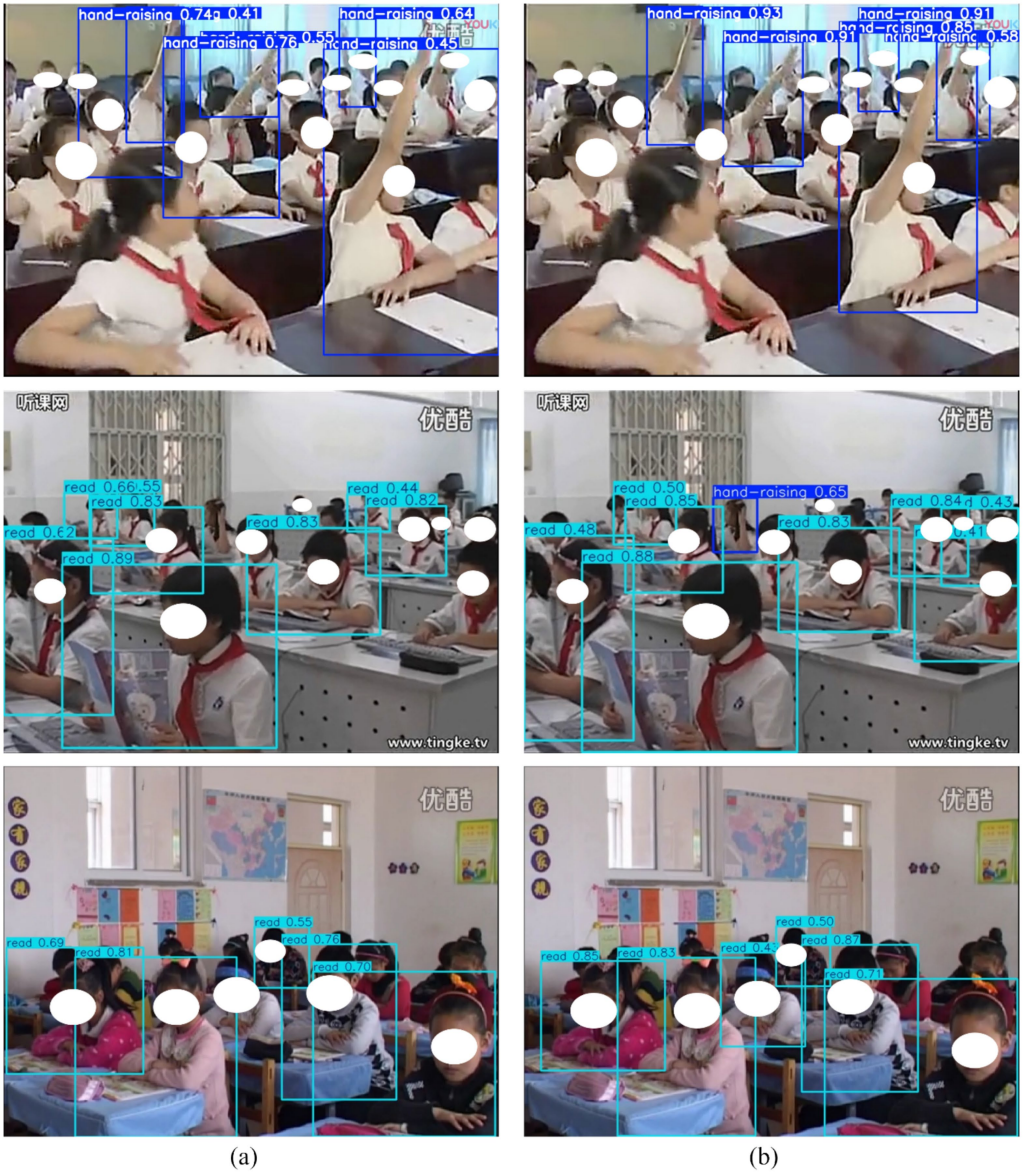
Visualization of detection results under challenging scenarios: **(a)** Detection results of the YOLOv8n baseline; **(b)** Detection results of SBR-YOLO.

Figure 6 presents qualitative comparisons between YOLOv8n and SBR-YOLO under three representative challenging scenarios in real classroom environments.

In dense distribution scenarios, inter-student proximity poses significant localization challenges. The baseline model produces overlapping bounding boxes and localization errors when students sit closely together. SBR-YOLO employs ASFF to adaptively aggregate multi-scale features, enabling precise boundary delineation through fine-grained spatial resolution at lower pyramid levels.

In severe occlusion cases, partial visibility caused by furniture or neighboring students leads to incomplete detections in the baseline model. SBR-YOLO employs BCPA to model global contextual dependencies, enabling the network to infer complete target regions from visible body parts through learnable positional encoding.

In behavior confusion scenarios, the baseline model misclassifies visually similar behaviors such as reading and writing due to insufficient inter-class discriminability. SBR-YOLO achieves correct classification through discriminative embeddings learned via CADL, which enforces intra-class compactness and inter-class separation constraints in the feature space.

### 4.10 Feature visualization and interpretability

**Figure 7:**
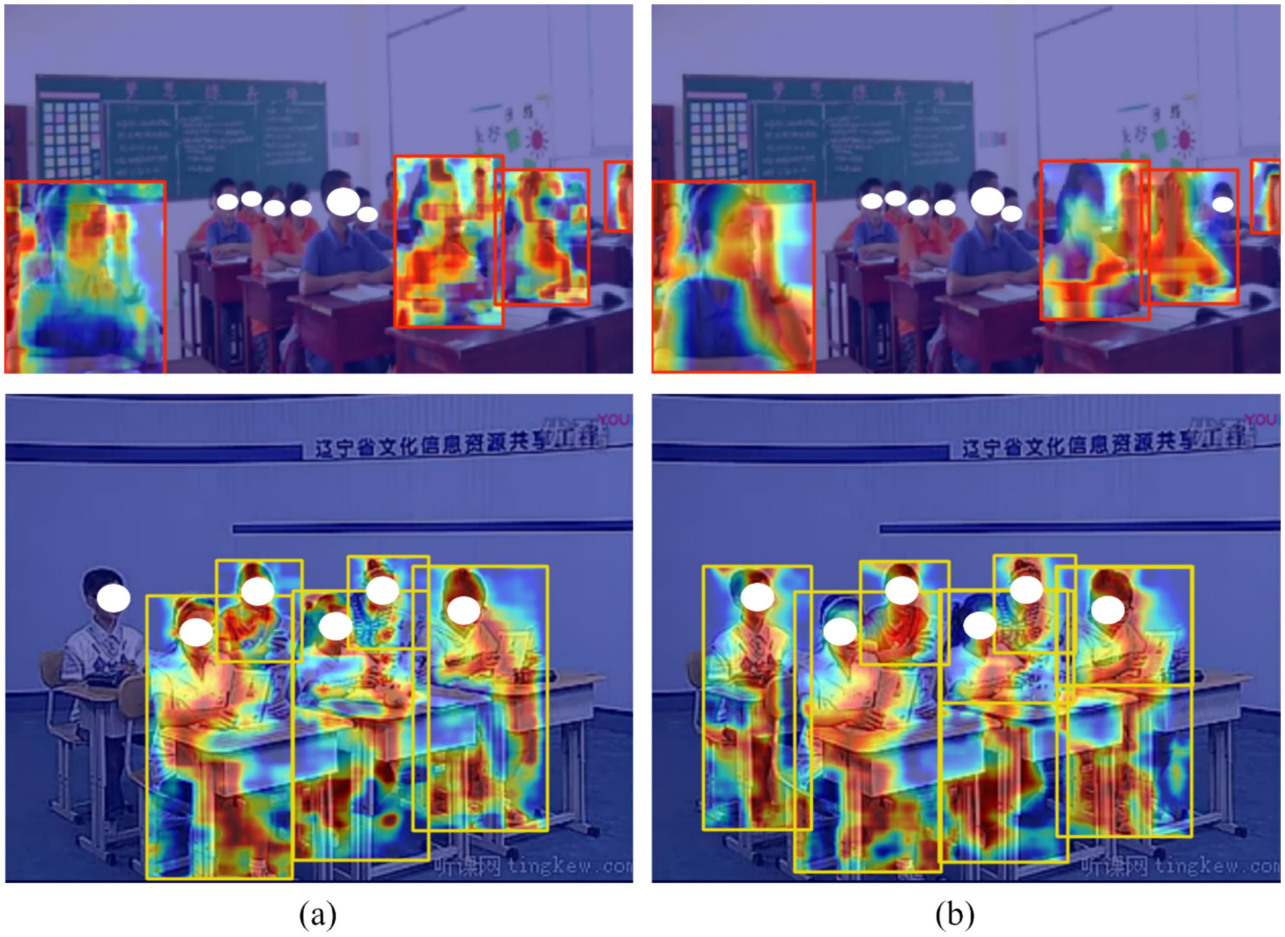
Comparative visualization of Grad-CAM class activation maps: **(a)** Baseline YOLOv8n; **(b)** Proposed SBR-YOLO.

Grad-CAM is applied to visualize the class activation maps of both the baseline model and SBR-YOLO. Figure 7 presents the corresponding activation maps. The baseline model exhibits a dispersed activation pattern across the image, indicating insufficient feature discriminability and leading to missed detections and false positives. In contrast, SBR-YOLO demonstrates concentrated activation on behavior-relevant regions. The activation maps reveal precise spatial localization aligned with the ground-truth target areas. This focused activation distribution confirms that the BCPA module guides the network to extract discriminative features from semantically meaningful regions, enhancing both localization accuracy and classification performance.

## 5 Conclusion

SBR-YOLO is proposed to address the challenges of fine-grained behavior recognition in complex classroom environments, where high intra-class variance and subtle inter-class differences among student behaviors impose significant demands on detection discriminability. Detection results can assist educators in identifying student engagement levels and learning states, enabling timely instructional adjustments.

SBR-YOLO comprises three core components: BCPA, ASFF, and CADL. BCPA leverages learnable positional encoding and inter-head interaction mechanisms to capture spatial dependencies among behavioral regions, enabling the network to focus on discriminative posture features while suppressing background interference. ASFF adaptively fuses multi-scale features through learned fusion weights, improving detection performance for student targets with substantial scale variations between front-row and back-row seating positions. CADL replaces CIoU in the detection heads by jointly optimizing geometric regression and feature-level discrimination, enforcing intra-class compactness and inter-class separation constraints to enhance discriminative capability for visually similar behaviors such as reading and writing without incurring additional inference cost.

Ablation experiments confirm that all three components contribute to improving detection accuracy. The model achieves an mAP@50 of 74.2% on SCB-Dataset3, representing a 6.4 percentage point improvement over the YOLOv8n baseline, with reduced rates of missed detections and false positives. These results validate the effectiveness of SBR-YOLO for student behavior recognition tasks.

The proposed framework addresses student behavior recognition in smart classroom environments, though several limitations warrant acknowledgment. First, experimental evaluation is restricted to SCB-Dataset3, a single benchmark comprising only three behavior categories, which constrains the assessment of cross-dataset generalizability and category scalability. Second, the framework operates on individual frames without incorporating temporal modeling, limiting its capacity to recognize dynamic behaviors that require motion context across consecutive frames. Third, detection accuracy under extreme imaging conditions, including severe occlusion and long-distance capture, remains suboptimal. Future work will focus on extending behavior category coverage, incorporating temporal sequence modeling and skeleton-based action recognition for dynamic behavior analysis, and exploring lightweight architecture designs to improve deployment efficiency across diverse educational scenarios.

## Data Availability

The original contributions presented in the study are included in the article/Supplementary material, further inquiries can be directed to the corresponding author.
